# Genetic mapping identified major main‐effect and three co‐localized quantitative trait loci controlling high iron and zinc content in groundnut

**DOI:** 10.1002/tpg2.20361

**Published:** 2023-07-05

**Authors:** Sejal Parmar, Pasupuleti Janila, Sunil S. Gangurde, Murali T. Variath, Vinay Sharma, Deekshitha Bomireddy, Surendra S. Manohar, Rajeev K. Varshney, Prashant Singam, Manish K. Pandey

**Affiliations:** ^1^ International Crops Research Institute for the Semi‐Arid Tropics (ICRISAT) Hyderabad India; ^2^ Department of Genetics Osmania University Hyderabad India; ^3^ Centre for Crop and Food Innovation, Food Futures Institute Murdoch University Murdoch Western Australia Australia

## Abstract

Malnutrition is a major challenge globally, and groundnut is a highly nutritious self‐pollinated legume crop blessed with ample genomic resources, including the routine deployment of genomic‐assisted breeding. This study aimed to identify genomic regions and candidate genes for high iron (Fe) and zinc (Zn) content, utilizing a biparental mapping population (ICGV 00440 × ICGV 06040;). Genetic mapping and quantitative trait locus (QTL) analysis (474 mapped single‐nucleotide polymorphism loci; 1536.33 cM) using 2 seasons of phenotypic data together with genotypic data identified 5 major main‐effect QTLs for Fe content. These QTLs exhibited log‐of‐odds (LOD) scores ranging from 6.5 to 7.4, explaining phenotypic variation (PVE) ranging from 22% (*qFe‐Ah01*) to 30.0% (*qFe‐Ah14*). Likewise, four major main effect QTLs were identified for Zn content, with LOD score ranging from 4.4 to 6.8 and PVE ranging from 21.8% (*qZn‐Ah01*) to 32.8% (*qZn‐Ah08*). Interestingly, three co‐localized major and main effect QTLs (*qFe‐Ah01, qZn‐Ah03, and qFe‐Ah11*) were identified for both Fe and Zn contents. These genomic regions harbored key candidate genes, including zinc/iron permease transporter, *bZIP transcription factor*, and *vacuolar iron transporter which likely play pivotal roles in the accumulation of Fe and Zn contents in seeds*. The findings of this study hold potential for fine mapping and diagnostic marker development for high Fe and Zn contents in groundnut.

## Core Ideas

- The authors identified nine major main effect and two main effect QTLs for Fe and Zn content.
- Three co‐localized QTLs for Fe and Zn contents were detected.
- Genomic regions harbored key candidate genes.

**Abbreviations**
μL: microliter
AhGEA: *Arachis hypogaea* gene expression atlas
bHLH: basic/helix‐loop‐helix
bZIP: basic region/leucine zipper motif
DNA: deoxyribonucleic acid
E‐QTLs: epistatic QTLs
Fe: iron
GB: gigabyte
GWAS: genome‐wide association studies
ICIM: inclusive composite interval mapping
ICP‐OES: inductively coupled plasma optical emission spectrometry
LEA: late embryogenesis abundant
LOD: Log‐of‐odds
NAC: no apical meristem
ppm: parts per million
PVE: phenotypic variation explained
QTL: quantitative trait locus;
RF: recombination frequencies
RIL: recombinant inbred line
RNA: ribonucleic acid
rpm: revolutions per minute
S1: first season
S2: second season
SNP: single‐nucleotide polymorphism
VIT: vacuolar iron transporter
ZIP: zinc/iron permease
Zn: zinc
*χ* ^2^
: chi‐square test

## 1 INTRODUCTION

Iron (Fe) and Zinc (Zn) are important micronutrients essential for human growth and development, as they serve as cofactors for several proteins, such as hemoglobin and cytochromes. More than three billion people suffer from iron (Fe) deficiencies worldwide (WHO, 2016). According to estimates, more than 60% of the world's population experiences anemia due to Fe and Zn deficiencies, commonly referred to as “hidden hunger” or malnutrition. In India, anemia caused by Fe deficiency affects 74% of children (6–35 months), 52% of nonpregnant women, and 80% of pregnant women (International Development Association). Symptoms of Fe and Zn deficiencies include dwarfism, tissue hypoxia, anemia, impaired disease immunity, poor cognitive development, stunning, and atopic dermatitis of the orifice and cranium (Bailey et al., 2015; Parmar et al., 2022; Tulchinsky, 2010).

Groundnut, also known as peanut (*Arachis hypogea* L.), is a nutritious self‐pollinated legume crop having ∼2.7 GB genome size. It is cultivated in >100 countries of arid and semiarid regions, covering a global cultivation area of 32.7 million hectares and yielding 53.92 million tons during 2021 (FAOSTAT, 2021). It is one of the richest sources of energy components, such as protein (25%–28%), oil (48%–50%), and carbohydrates (10%–20%), in the seeds. They also contain monounsaturated fatty acids, vitamins, minerals, and antioxidants. Groundnut seeds are abundant in several B‐complex vitamins and minerals (thiamin, pantothenic acid, vitamin B6, folates, and niacin), and antioxidants like *p*‐coumaric acid and resveratrol. Moreover, groundnuts contain significant amounts of bioactive polyphenols, flavonoids, and isoflavones. Due to their high nutritional content, groundnut and products made from them can be marketed as nutritious foods, addressing protein, energy, and micronutrient deficiencies in impoverished populations. Groundnut‐based ready‐to‐use therapeutic products, such as plumpy nuts, have saved the lives of undernourished children in Niger (UNSCN, 2007).

Now, groundnut has optimum genomic resources, including reference genomes, diagnostic markers, and genotyping assays (Pandey et al., 2020). The availability of genome‐wide markers and genotyping assays is important for implementing genomics and breeding activities, aimed at enhancing nutritional components. High‐to‐mid‐density genotyping arrays/assays are important for constructing genetic maps, identifying the genomic regions and candidate genes through genetic mapping and genome‐wide association studies (GWAS), and developing diagnostic markers for breeding programs. In groundnut, trait‐associated single‐nucleotide polymorphism (SNP) markers have been developed and validated for many traits, such as fresh seed dormancy (Bomireddy et al., 2022; Kumar et al., 2020), foliar disease resistance (Pandey et al., 2017), high oleic acid (Chu et al., 2009), seed size (Gangurde et al., 2023; Zhuang et al., 2019), leaf spot and tomato spotted wilt virus resistance (Agarwal et al., 2019, 2018), shelling percentage (Luo et al., 2019a), and bacterial wilt resistance (Luo et al., 2019b).

**Core Ideas**

- The authors identified nine major main effect and two main effect QTLs for Fe and Zn content.
- Three co‐localized QTLs for Fe and Zn contents were detected.
- Genomic regions harbored key candidate genes.

Several reports have documented quantitative trait locus (QTL) mapping for the seed Fe and Zn content in legumes and cereals, including chickpeas (Sab et al., 2020), pearl millet (Kumar et al., 2018), rice (Dixit et al., 2019; Verma et al., 2022), wheat (Crespo‐Herrera et al., 2017), maize (Vemuri et al., 2018), and soybean (Wang et al., 2022). However, in groundnut, only a few studies have been conducted, using single marker analysis with mere 33 markers (Kurapati, 2015) and GWAS with single sequence repeats and diversity arrays technology markers (Pandey et al., 2014) for Fe and Zn contents. Unfortunately, these studies were inconclusive due to the lack of high‐throughput genotyping assays aligned with a physical position in the tetraploid groundnut genome. Nevertheless, a few phenotyping studies have confirmed genetic variation for Fe and Zn contents in groundnut seeds. Significant variations between genotypes and environments were reported for Fe (33–68 mg/kg) and Zn (44–95 mg/kg) in groundnut seeds (Janila et al., 2015), which can be leveraged to develop breeding strategies aimed at increasing Fe and Zn contents in groundnut. This study also identified two genotypes (ICGV 06099 and ICGV 06040) that consistently exhibited high Fe and Zn contents. In a cross, ICGV 06040 × ICGV 87141, generation mean analysis showed that additive gene action played a predominating role in controlling the Fe and Zn contents. In contrast, the cross ICGV 06099 × ICGV 93468 showed both additive and additive × additive interactions. Cotyledons contribute approximately 85%–90% of the total Fe and Zn contents on a dry matter basis, with the remaining 10%–15% in present in the embryo and seed coat of groundnut seeds (Kurapati et al., 2021).

In plants, two strategies (strategies I and II) regulate iron uptake from the rhizosphere (Connorton, Balk, et al., 2017). Legumes follow strategy I, which involves acidifying the rhizosphere to increase Fe^+3^ solubility (Roorkiwal et al., 2021). Several putative homologs involved in transporting Fe from the leaf to the root, such as *FIT1*, *IRT1*, *OPT3*, and *bZIP23*, have been identified in legumes, including peanuts (Xiong et al., 2012). Zn is mainly absorbed by the plasma membrane of root cells as Zn^+2^, and zinc/iron permease (*ZIP*) *transporters* are responsible for the uptake and transport of Zn from root to seeds (Colangelo & Guerinot, 2006; Palmagren et al., 2008). There are many genes or gene families regulating iron and zinc homeostases, such as *YSL*, *NRAMP*, *ZIP*, *Heavy metal ATPase*, *cation diffusion facilitator family*, *nicotianamine synthase*, and *nicotianamine aminotransferase*, which have been reported in cereals (Anuradha et al., 2012). The primary objective of the present study was to identify the QTLs associated with Fe and Zn contents, as well as to determine the candidate genes responsible for the accumulation of Fe and Zn in groundnut seeds. In this context, a recombinant inbred line (RIL) population derived from the cross between ICGV 00440 (with low Fe and Zn contents) and ICGV 06040 (with high Fe and Zn contents) was developed. This population was genotyped using a 5K mid‐density assay, and phenotyping was carried out using inductively coupled plasma optical emission spectrometry (ICP‐OES) spectroscopy for Fe and Zn contents over two seasons. Genetic mapping identified major main effect QTLs and candidate genes for accumulating Fe and Zn contents in groundnut seeds. QTL regions can be utilized to develop diagnostic markers, which can then be employed to select breeding lines with high Fe and Zn contents.

## 2 MATERIAL AND METHODS

### 2.1 Development of RIL population and phenotyping for Fe and Zn contents

A biparental mapping population (ICGV 00440 × ICGV 06040) comprising 218 RILs was developed and advanced by a single seed descent method. The donor parent, ICGV 06040, has significantly higher Fe and Zn contents than the recipient parent, ICGV 00440. ICGV 06040 is a Spanish bunch type, medium duration, and high Fe and Zn content parent; on the other hand, ICGV 00440 has low Fe and Zn contents. The experiment was conducted at ICRISAT, Patancheru, India during two seasons post‐rainy 2018–19 (S1) and rainy 2019 (S2). However, the field experiment was designed in an alpha lattice design with 4 m rows on points 30 cm apart. In the soil sample, Fe and Zn contents were 5.46 and 2.98 ppm, respectively. A RIL population ICGV 00440 **×** ICGV 06040 was phenotyped at ICRISAT, Patancheru, Hyderabad (India) during S1 and S2.

Fe and Zn contents were estimated using the ICP‐OES method (Prodigy High Dispersion ICP, TELEDYNE Leeman Labs, USA) (Wheal et al., 2011). About ∼0.3 g of oven‐dried samples were used and transferred into 50 mL labeled polypropylene tubes. Nitric acid of 2 mL was added, followed by 0.5 mL hydrogen peroxide in a tube using a bottle top dispenser, and then allowed to stand overnight at room temperature. Next day, tubes were vortexed once more before being put into the digestion block following the protocol. The samples were initially allowed to digest at 80°C for 30 min before being kept at 125°C for 120 min. After the initial 30 min of warming, pressure built up in the tubes was released by sliding each cap just enough to equalize the pressure. Once the program was complete, the digested samples were taken out of the digestion block and chilled to room temperature; no additional handling was done. Distilled water was made up to a volume of 25 mL and then mixed on an orbital mixer or in a vortex for 5 min. Then samples were filtered, and the supernatant was taken out for analysis on ICP.

Two seasons of phenotyping data for Fe and Zn contents (ppm) were generated for the RIL population and parental lines. Frequency distribution graphs (violin plots) of Fe and Zn contents were generated using the “vioplot” package in R software. However, the Pearson correlation plot was generated using “metan” package in R software.

### 2.2 DNA extraction and genotyping with 5K mid‐density genotyping assay

Genomic deoxyribonucleic acid (DNA) was extracted using a 100 mg leaf sample of each line in the RIL population, including both parents using the Nucleospin Plant II kit (Macherey‐Nagel, Duren, Germany; https://guest.link/UM6). The leaf tissue of 100 mg was homogenized using 500 μL of lysis buffer. To remove ribonucleic acid (RNA) impurities, 10 μL RNAse was used. The samples were incubated at 65°C for 1 h in water bath. The samples were centrifuged for 10 min at 5500 rpm. The supernatant was collected in a separate tube, and a 450 μL binding buffer was added. This mixture was filtered using the Nucleospin plant MN column. The column was centrifuged at 6000 rpm for 1 min. As a final step, 50 μL warm elution buffer was added to the membrane filter of a column. The mixture was then incubated for 5 min at 65°C and centrifuged for 1 min at 6000 rpm to elute the DNA. The quality of DNA was checked on 0.8% agarose gel, whereas the quantity of DNA was checked using NanoDrop 8000 Spectrophotometer (Thermo Fisher Scientific Inc., Waltham, MA, USA). DNA 20 μL of with a concentration of 20 ng/μL from each sample was sent to Thermo Fisher's AgriSeq Targeted genotyping by sequencing platform, USA for genotyping using a mid‐density 5K SNP assay including two parents. Each SNP was named chromosome number followed by its physical position on tetraploid reference genome, for example, Ah01_57714; here, Ah01 was chromosome number, and the “57714” was the position of SNP on that chromosome. The chi‐square test (*χ* ^2^) was used to determine each SNP marker's segregation ratio (1:1). However, distorted markers were filtered out to keep the good quality of genetic map.

### 2.3 Construction of genetic linkage map

A genetic map was constructed using the QTL IciMapping software version 4.2 (Wang et al., 2019). Markers were grouped by applying a recombination frequency (RF) (*∂*) threshold of 30% followed by ordering and rippling. The genetic linkage map with appropriate map length was constructed by deleting, adding, and rearranging the markers by functions in IciMapping software. Kosambi map function was used to convert RF into map distances centiMorgans (cM) (Kosambi, 1943). Allelic calls for each SNP were noted based on parents; the female (recurrent) parent ICGV 00440 type calls (AA) were symbolized as 2, male (donor) parent ICGV 06040 type calls (BB) were symbolized as “0,” whereas heterozygotes were symbolized as “1” and “−1” for missing calls.

### 2.4 QTL analysis for Fe and Zn contents

Two seasons’ (S1 and S2) phenotypic data, genetic map information, and genotyping data were used to identify the QTLs associated with Fe and Zn contents. Main effect QTLs were identified using inclusive composite interval mapping (ICIM) additive method in Windows QTL cartographer V2.5 011 (Wang et al., 2011), and epistatic QTLs (E‐QTLs) were identified using ICIM epistatic method implemented in IciMapping software v4.1.0.0. The scanning step in ICIM was set to 1.0 cM, and the *p*‐values for entering variables (PIN) and eliminating variables (POUT) were set to 0.001 and 0.002, respectively. The presence of a QTL was determined using a log‐of‐odds (LOD) threshold of 3.0. QTLs with more than 10% of phenotypic variance were considered major main effect QTLs; however, QTLs with less than 10% phenotypic variance were considered main effect QTLs. QTLs are annotated by a lowercase *q*, followed by shortened capital letters that denote the trait and chromosomal number. For instance, *qFe‐Ah01* denotes the first QTL for iron on chromosome Ah01. The linkage map and QTLs were constructed using Map Chart version 2.3 for visualization of QTLs’ respective positions genetic map.

### 2.5 Identification of candidate genes and expression analysis for Fe and Zn contents

Candidate genes were mined from identified QTLs for Fe and Zn contents. Genomic region between the physical positions of the flanking markers of each QTL was used to study the candidate genes in peanut base (https://Peanutbase.org/) through GBrowse (cultivated peanut) version 1. *Arachis hypogaea* gene expression atlas (*AhGEA*) generated on ICGV 91114 ssp. *fastigiata* (Sinha et al., 2020) was used to check the expression of candidate genes. With the help of gene expression atlas, expression data was accessed for 20 tissues for identified candidate genes for Fe and Zn contents.

## 3 RESULTS

### 3.1 Phenotypic variation for Fe and Zn contents in RIL population

Two seasons of phenotyping data were generated during S1 and S2. The parental genotype, ICGV 06040, with high Fe and Zn contents, was used as a donor parent, whereas ICGV 00440, with low Fe and Zn contents, was used as a recipient parent. ICGV 06040 contained 50.5 and 54.3 ppm Fe content and 65.5 and 60.3 ppm Zn content during S1 and S2, respectively. ICGV 00440 contained 30.5 and 32.5 ppm Fe content and 33.3 and 35.7 ppm Zn content during S1 and S2, respectively.

During first season (S1), Fe content ranged from 19.40 to 47.92 ppm in the population. Although the higher value was 47.92 ppm, the lower value was 19.40 ppm, and the mean value was 30.83 ppm. However, second season (S2) Fe content ranged from 22.2 to 80.79 ppm. The higher value was 80.79 ppm, lower value was 22.51 ppm, and the mean value was 31.62 ppm.

**FIGURE 1:**
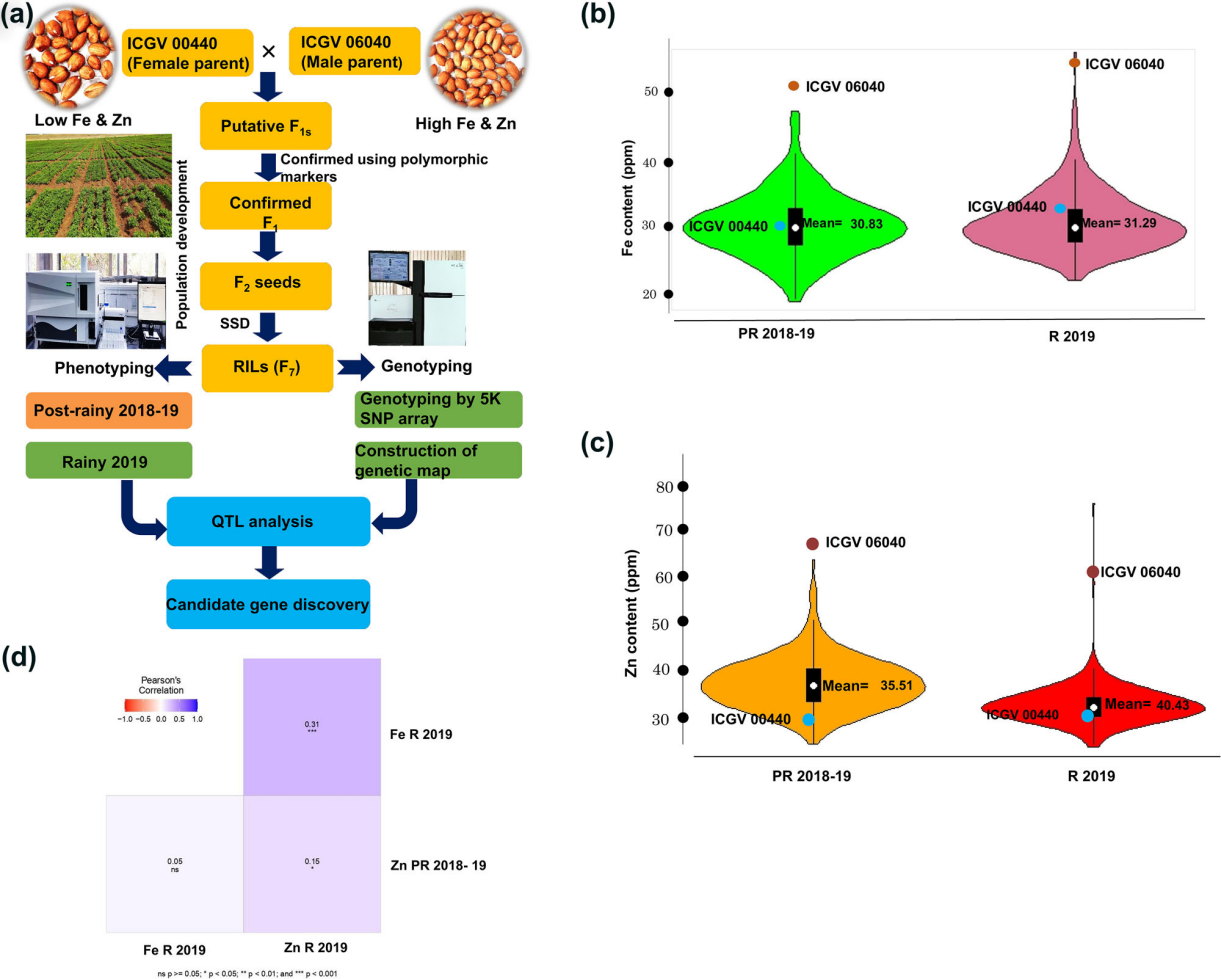
Population development, phenotyping and genotyping of recombinant inbred line (RIL) population. The figure shows (a) flowchart regarding development, genotyping and phenotyping of RIL population for Fe and Zn content; (b) frequency distribution of phenotypic data generated for Fe content in RIL population, in that brown dot shows parental genotype ICGV 06040 and blue dot shows parental genotype ICGV 00440. Similarly, the figure (c) shows frequency distribution of phenotypic data generated for Zn content in RIL population in that brown dot shows parental genotype ICGV 06040 and blue dot shows parental genotype ICGV 00440, and plot (d) correlation plot shows the correlation of Fe and Zn content.

Zn content during S1 ranged from 27.14 to 78.86 ppm. Although the higher value was 78.86 ppm, the lower value was 27.14 ppm, and the mean value was 35.51 ppm. During S2, Zn content ranged from 26.6 to 61.74 ppm. The higher value was 61.74 ppm, the lower value was 26.6 ppm, and the mean value was 40.43 ppm. In both seasons, Zn content was higher than the Fe content (Figure 1). Additionally, the violin plots to visualize the frequency distribution for the Fe and Zn contents showed wide variation. Furthermore, the Pearson correlation (*r*) analysis revealed strong positive correlation (*r* = 0.31) between Fe and Zn contents in S2. However, during S1, there was no significant correlation between Fe and Zn contents (Figure 1).

### 3.2 Construction of genetic linkage map

**FIGURE 2:**
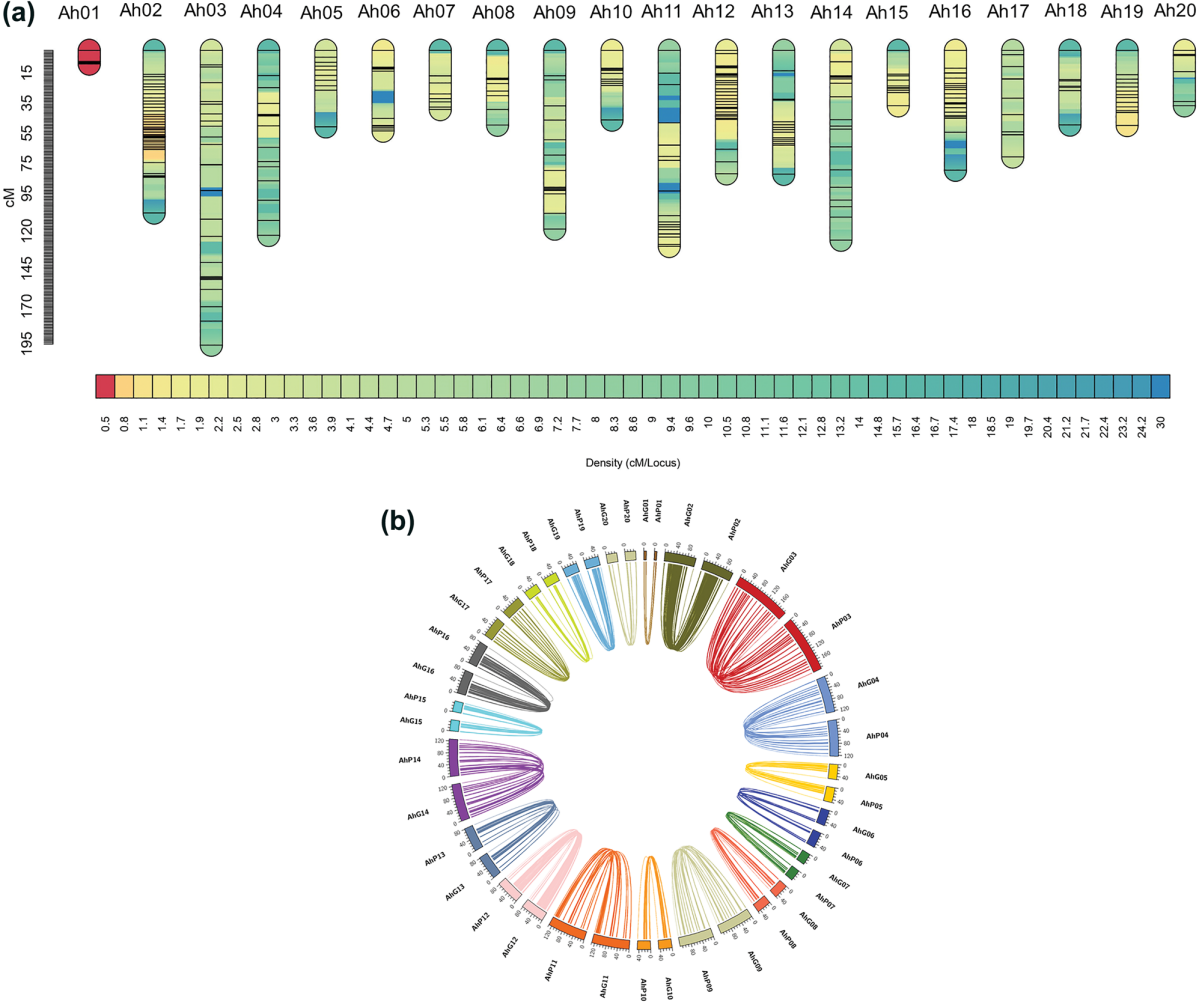
Features of the genetic map of recombinant inbred line (RIL) population. The figure shows (a) map chart of genetic map for RIL population and (b) collinearity of the genetic map and physical map for RIL population.

Genotyping data was generated from 5K SNP assay for 218 RILs and parental genotypes. Of the 5078 SNPs, 4346 were found monomorphic between the parents, and 248 SNPs were missing in both parents. Finally, after rigorous filtration, 571 polymorphic SNPs were obtained and used to construct a genetic map. Of the 571 SNPs, 97 were distorted or not grouped with linkage groups. A genetic linkage map was constructed, comprising a total of 474 SNP loci, and spanned a map length of 1536.33 cM. The length of individual linkage groups ranged from 8.99 cM (Ah01) to 195.74 cM (Ah03). The average inter‐marker distance per individual linkage group varied from 0.4 (Ah01) to 4.9 (Ah18). The highest number of SNPs were mapped on Ah02 with 63 SNPs, with 107.92 cM map length and 1.5 cM average marker distance (Table 1; Figure 2).

**TABLE 1:** Summary of genetic map constructed for recombinant inbred line (RIL) population.

Linkage group	Total polymorphic loci	Total mapped loci	Map distance (cM)	Average marker distance (cM)
Ah01	21	19	8.99	0.42
Ah02	68	63	107.92	1.58
Ah03	43	39	195.74	4.55
Ah04	29	24	122.82	4.23
Ah05	15	14	50.69	3.37
Ah06	32	18	53.47	1.67
Ah07	18	14	39.20	2.17
Ah08	21	19	49.56	2.36
Ah09	34	28	118.57	3.48
Ah10	15	14	46.17	3.07
Ah11	36	29	130.12	3.61
Ah12	52	45	81.93	1.57
Ah13	34	22	82.21	2.41
Ah14	29	26	125.96	4.34
Ah15	21	15	36.74	1.74
Ah16	35	27	79.52	2.27
Ah17	20	15	70.71	3.53
Ah18	10	10	49.49	4.94
Ah19	28	23	49.83	1.77
Ah20	10	10	36.69	3.66
**Total**	**571**	**474**	**1536.33**	**2.83**

### 3.3 QTLs for Fe and Zn contents

A total of 11 main‐effect QTLs were identified for Fe and Zn contents; among them, 9 were major main‐effect QTLs and 2 were main‐effect QTLs.

For the seed Fe content, six QTLs, namely, *qFe_Ah01, qFe_Ah03, qFe_Ah07, qFe_Ah04, qFe_Ah14*, and *qFe_Ah11*, were identified on chromosome Ah01, Ah03, Ah07, Ah04, Ah14, and Ah11, respectively. Their LOD scores ranged from 3.9 to 7.3 during S2, and phenotypic variation explained (PVE) ranged from 7.3% to 30%. The favorable allele for QTL (*qFe_Ah14*) was contributed by ICGV 00440, whereas ICGV 06040 contributed the favorable allele for QTLs (*qFe_Ah01, qFe_Ah03, qFe_Ah07, qFe_Ah04*, and *qFe_Ah11*).

**FIGURE 3:**
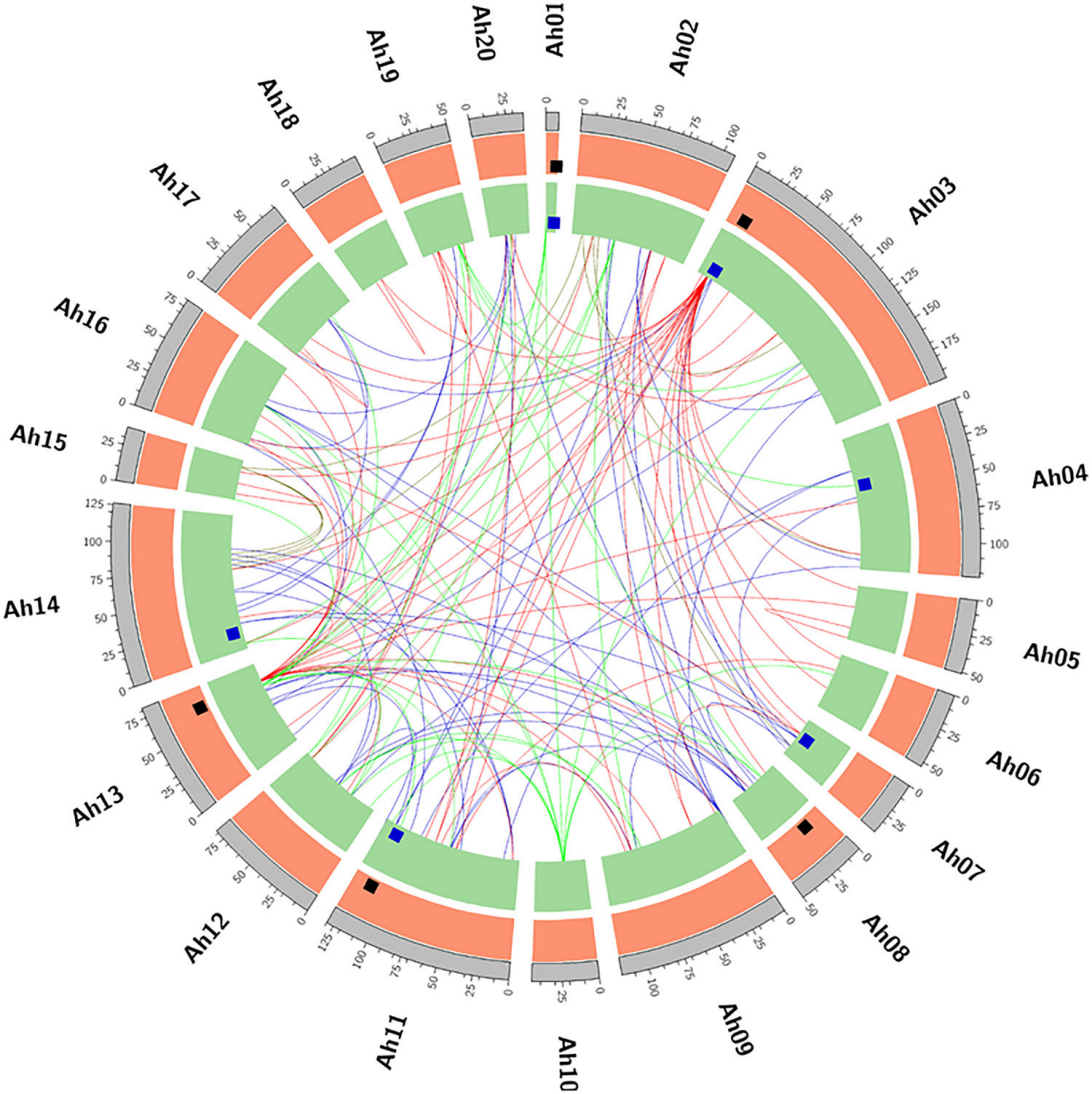
Major main‐effect quantitative trait loci (QTLs) and epistatic QTLs for Fe and Zn contents for two seasons. Blue block representing major main effect QTLs for Fe content and black block representing major main effect QTLs for Zn content.

A total of five QTLs (*qZn_Ah01, qZn_Ah03, qZn‐Ah08, qZn‐Ah13*, and *qZn‐Ah11*) were identified for seed Zn content on chromosomes Ah01, Ah03, Ah08, Ah13, and Ah11, respectively, with LOD score ranging from 3.2 to 6.8 and PVE ranged from 5.62% to 32.78%. Four QTLs (*qZn_Ah01, qZn_Ah03, qZn‐Ah08*, and *qZn‐Ah13*) were identified in S1 with 4.4–6.8 LOD scores and 21.8%–32.8% PVE. One QTL (*qZn‐Ah11*) was identified in S2 with 3.2 LOD score and 5.2% PVE. The favorable alleles for four QTLs (*qZn_Ah01, qZn_Ah03, qZn‐Ah08*, and *qZn‐Ah11*) were contributed by ICGV 06040, whereas ICGV 00440 contributed the favorable allele for QTL *(qZn‐Ah13)*. It is interesting to note that we found three co‐localized genomic regions for seed Fe and Zn contents, that is, *qFe_Ah01* during *S2* and *qZn_Ah01* during S1*, qFe_Ah03* during S2 and *qZn_Ah03* during S1, and *qFe_Ah11 and qZn‐Ah11* during S2. All co‐localized QTLs for Fe and Zn contents had positive additive effect with favorable alleles from the ICGV 06040 (Table 2; Figure 3).

**TABLE 2:** Summary of main‐effect quantitative trait loci for seed Fe and Zn contents in recombinant inbred line (RIL) population.

QTL	Season	Chromosome	Left marker	Right marker	Position (cM)	LOD	PVE (%)	Additive effect
*qFe_Ah14*	S2	Ah14	Ah14_20446682	Ah14_134726415	15.52	7.4	30	−7.6
*qFe_Ah03*	S2	Ah03	Ah03_143428046	Ah03_136234652	11.97	4.1	27.7	8.1
*qFe_Ah04*	S2	Ah04	Ah04_124480054	Ah04_77545317	44.47	4.3	27.4	5.5
*qFe_Ah07*	S2	Ah07	Ah07_63803442	Ah07_573492	16.01	7.2	24.9	10.8
*qFe_Ah01*	S2	Ah01	Ah01_57714	Ah01_2239967	7.01	6.5	22	6.6
*qFe_Ah11*	S2	Ah11	Ah11_134910677	Ah11_104708625	114.9	4	7.3	1.5
*qZn_Ah08*	S1	Ah08	Ah08_37701095	Ah08_51688050	16.01	5.8	32.8	8.4
*qZn_Ah03*	S1	Ah03	Ah03_143428046	Ah03_136234652	11.97	6.8	27.3	12.6
*qZn_Ah13*	S1	Ah13	Ah13_ 33322684	Ah13_137038170	64.84	4.4	26.5	−10.8
*qZn_Ah01*	S1	Ah01	Ah01_57714	Ah01_2239967	7.01	4.7	21.8	8.4
*qZn_Ah11*	S2	Ah11	Ah11_134910677	Ah11_104708625	113.9	3.2	5.6	1.1

Abbreviations: LOD, log‐of‐odds; PVE (%), phenotypic variation explained; QTL, quantitative trait locus; S1, season 1; S2, season 2.

### 3.4 Identification of epistatic QTLs (E‐QTLs) for Fe and Zn contents

Total of 142 E‐QTLs were identified for Fe and Zn contents. Among them, 55 and 85 major E‐QTLs were identified for Fe and Zn contents with 15.43%–80.43% PVE.

For seed Fe content, 47 major E‐QTLs were identified in S1, with LOD scores ranging from 40.17 to 67.21 and PVE ranging from 79.45% to 80.31%. In S2, eight major E‐QTLs were identified with LOD scores ranging from 7.02 to 9.2 and PVE ranging from 17.91% to 28.02%. For Zn content, 55 major E‐QTLs were identified in S1, with LOD score 8.75–7.01 and 34.60%–17.92% PVE. However, 30 major E‐QTLs were identified in S2 with LOD scores of 8.15–7.0 and 30.94%–15.43% PVE.

Interestingly, co‐localized major main effect QTLs, namely, *qFe_Ah01* and *qZn_Ah01* with 7.6 LOD and 15.6% PVE, as well as *qFe_Ah03 and qZn_Ah03* with 61.6 LOD and 79.5% PVE, were also observed in epistatic interaction (Table S1; Figure 3).

### 3.5 Candidate genes for Fe and Zn contents in co‐localized QTL regions

**FIGURE 4:**
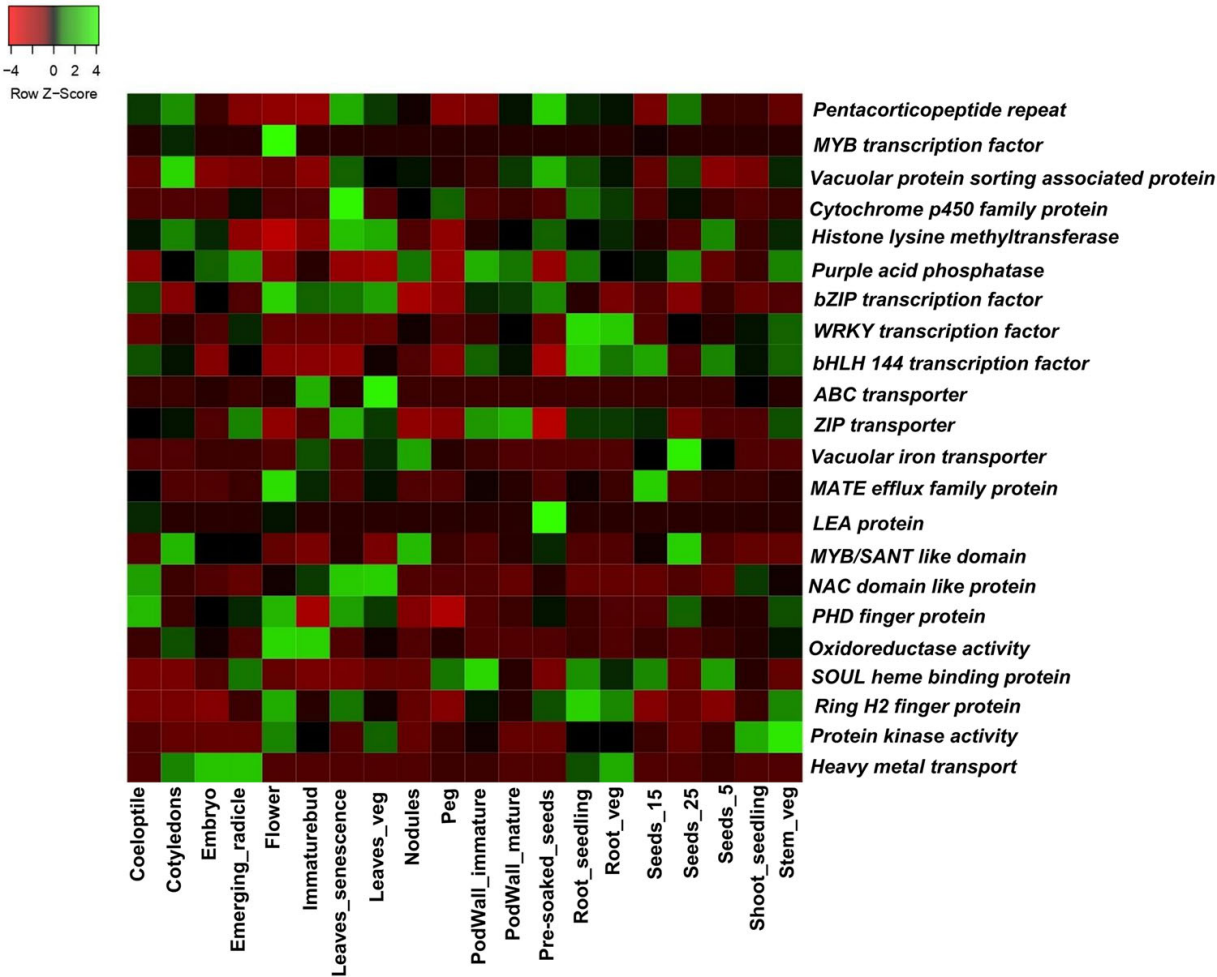
Tissue‐specific expression of candidate genes identified in quantitative trait locus (QTL) regions for Fe and Zn contents. Expression of candidate genes was studied using *Arachis hypogaea* gene expression atlas. The expression of 22 candidate genes in 20 tissues (Coeloptile, Cotyledons, Embryo, Emerging_radicle, Flower, Immature bud, Leaves_senescence, Leaves_veg, Nodules, Peg, PodWall_immature, PodWall_mature, Pre‐soaked_seeds, Root_seedling, Root_veg, Seeds_15, Seeds_25, Seeds_5, Shoot_seedling, Stem_veg) are plotted in the heatmap.

The genes associated with Fe and Zn contents were identified within the intervals of co‐localized and major QTLs. Most of these genes are known to be involved in the uptake, transportation, and accumulation of Fe and Zn in groundnut seeds. A total of 25 candidate genes were identified in co‐localized and other major main effect QTLs (Table 3). Among these, 20 candidate genes were found on co‐localized chromosome Ah01 *(qFe‐Ah01/qZn‐Ah01)*, Ah03 (*qFe‐Ah03/qZn‐Ah03*), and Ah11 (*qFe‐Ah11/qZn‐Ah11*). Additionally, five candidate genes were found on major QTLs *qZn‐Ah13, qFe‐Ah07*, and *qZn‐Ah08*. The tissue‐specific expression of these genes was studied using *fastigiata* subspecies, gene expression atlas (*AhGEA*) (Sinha et al., 2020). A heat map was generated, plotting the expression data of 22 candidate genes across 20 peanut tissues at various growth stages (Table S2; Figure 4).

**TABLE 3:** Candidate genes underlying quantitative trait loci (QTLs) for Fe and Zn contents in recombinant inbred line (RIL) population.

Traits	QTL name	Chromosome	Gene id	Functional annotation
Fe/Zn	*qFe‐Ah01/qZn‐Ah01*	Ah01	*arahy.X7MPPU*	*Pentacorticopeptide repeat*
		Ah01	*arahy.VE9WMU*	*MYB transcription factor*
		Ah01	*arahy.S59764*	*Vacuolar protein sorting associated protein*
		Ah01	*arahy.D92ENK*	*Cytochrome p450 family protein*
		Ah01	*arahy.UU22QK*	*Protein FAR1‐RELATED SEQUENCE 3*
		Ah01	*arahy.GI7SC3*	*Histone lysine methyltransferase*
		Ah01	*arahy.6AWP8P*	*WD40 repeat‐containing protein SMU1‐like*
		Ah01	*arahy.V6RGS7*	*Purple acid phosphatase*
Fe/Zn	*qFe‐Ah03/qZn‐Ah03*	Ah03	*arahy.AY0SR4*	*bZIP transcription factor*
		Ah03	*arahy.GB36NI*	*WRKY transcription factor*
		Ah03	*arahy.E4I2JR*	*bHLH 144 transcription factor*
		Ah03	*arahy.3YED3G*	*ABC transporter*
		Ah03	*arahy.31BYXQ*	*ZIP transporter*
		Ah03	*arahy.8KMA2D*	*Vacuolar iron transporter*
		Ah03	*arahy.42DQ0K*	*SAUR‐like auxin‐responsive protein family*
		Ah03	*arahy.Q5QJ3U*	*MATE efflux family protein*
Fe/Zn	*qFe‐Ah11/qZn‐Ah11*	Ah11	*arahy.QAC8GG*	*LEA protein*
		Ah11	*arahy.MH35TT*	*MYB/SANT like domain*
		Ah11	*arahy.JE37KP*	*NAC domain like protein*
		Ah11	*arahy.KMR140*	*PHD finger protein*
Zn	*qZn‐Ah13*	Ah13	*arahy.H1181I*	*Oxidoreductase activity*
		Ah13	*arahy.4X60UW*	*SOUL heme binding protein*
Fe	*qFe‐Ah07*	Ah07	*arahy.1WFS8V*	*Ring H2 finger protein*
		Ah07	*arahy.VAAE0N*	*Protein kinase activity*
Zn	*qZn‐Ah08*	Ah08	*arahy.R4WH7M*	*Heavy metal transport*

Abbreviation: NAC, no apical meristem.

## 4 DISCUSSION

Micronutrient malnutrition is a major issue worldwide. To deal with the micronutrient malnutrition or hidden hunger, bio‐fortification is the key process to enhance the nutritional value of crops. Groundnut is an important source of nutrition providing an opportunity to improve its micronutrient content, such as Fe and Zn. Genomic‐assisted breeding has shown potential to improve crop varieties for multiple traits in groundnut. For instance, high oleic groundnut varieties developed using marker‐assisted breeding by introgression fatty acid desaturase gene (Janila et al., 2016; Shasidhar et al., 2020). However, only a few studies have been conducted on the QTL mapping and GWAS for Fe and Zn contents in groundnut, and the results were inconclusive due to the limitations of high‐throughput genotyping assays. In this study, we identified 11 main‐effect QTLs, including 3 co‐localized QTLs, and 25 candidate genes associated with Fe and Zn contents. Understanding the homeostasis pathway of Fe and Zn contents is crucial for the development of Fe‐ and Zn‐rich groundnut varieties using genomics‐assisted breeding.

The RIL population was evaluated for two seasons, and their Fe and Zn contents were measured using ICP‐OES spectroscopy (Wheal et al., 2011). The RIL population and parents exhibited wide variation in Fe and Zn contents. Several studies have reported variations in Fe and Zn contents in crops, such as maize (Maziya‐Dixon et al., 2000), rice (Anuradha et al., 2012), sorghum (Kumar et al., 2009), soybean (Wang et al., 2022), and groundnut (Upadhyaya et al., 2012; Janila et al., 2014). Understanding the correlation between quantitative traits, such as Fe and Zn contents, can facilitate the pyramiding of multiple traits in breeding. In this study, Fe a significant positive correlation was observed between Fe and Zn contents in the RIL population for one season. Previous studies in groundnut (Janila et al., 2015; Upadhyaya et al., 2012) and other cereals and legume crops, including wheat (Badkhshan et al., 2013; Ghanbari & Mameesh, 1971; Velu et al., 2011), pearl millet (Govindaraj et al., 2009; Kanatti et al., 2014; Pujar et al., 2020), sorghum (Ravikiran et al., 2014; Susmitha & Selvi, 2014), rice (Bekele et al., 2013), and soybean (Wang H et al., 2022), have also reported a positive correlation for Fe and Zn contents. This indicates the involvement of common pathways in the homeostasis of the Fe and Zn contents.

SNP arrays offer a fast and efficient method for high‐throughput genotyping, which can be used for association analysis, diversity analysis, and QTL mapping. In groundnut, high‐density array and mid‐density SNP assays have been developed and employed in the genetic dissection of complex traits (Bomireddy et al., 2022). In this study, we constructed a genetic linkage map consisting of 474 loci, covering a map length of 1536.33 cM with an average inter‐marker distance of 2.83 cM per locus. This genetic linkage map was utilized to identify QTLs related to seed Fe and Zn contents. We identified a total of nine major main‐effect QTLs for Fe and Zn content, with five QTLs identified for Fe content on chromosomes Ah01, Ah03, Ah07, Ah14, and Ah04 with 22%–30% PVE and LOD ranging from 4.1 to 7.4. Similarly, for Zn content, four QTLs were detected on Ah01, Ah03, Ah08, and Ah13 with 21.8%–32.8% PVE and LOD ranged from 4.4 to 6.8. Previously, several studies have reported QTLs for seed Fe and Zn contents in various crops, such as chickpea (Sab et al., 2020), pearl millet (Kumar et al., 2018), rice (Dixit et al., 2019), wheat (Crespo‐Herrera et al., 2017), maize (Vemuri et al., 2018), and soybean (Wang et al., 2022). Throughout all of these studies, the QTLs were distributed on several chromosomes, which clearly indicates the quantitative nature and complexity of these traits. Interestingly, we have identified three co‐localized QTLs (*qFe‐Ah01/qZn‐Ah01, qFe‐Ah03/qZn‐Ah03*, and *qFe‐Ah11/qZn‐Ah11*) associated with Fe and Zn contents. Similar co‐localized QTLs for Fe and Zn contents have been detected in cereals and legumes, such as chickpeas (Sab et al., 2020), rice (Dixit et al., 2019), and wheat (Crespo‐Herrera et al., 2017). Presence of the strong linkage or pleiotropism of genes controlling several traits may explain the occurrence of co‐localized QTLs (Chen et al., 2016). Co‐localized QTLs are important for developing high Fe‐ and Zn‐containing varieties and for synchronously improving Fe and Zn contents in groundnut. Additionally, we have identified total of 142 E‐QTLs for Fe and Zn contents, out of which 55 and 85 major E‐QTLs were identified for Fe and Zn contents, respectively, with PVE ranging from 15.43% to 80.43%. Similar findings were reported in previous study on epistatic interaction for Fe and Zn contents in rice (Swamy et al., 2018),

Now, groundnut has made significant progress in genomic resources, enabling researchers to identify candidate genes and study tissue expression (Pandey et al., 2020). In our study, we identified 25 candidate genes in QTL regions that control Fe and Zn contents and are involved in the homeostasis of Fe and Zn contents in seeds. Among these 25 candidate genes, 22 showed expression during different developmental stages. Previous studies have highlighted the involvement of *bHLH transcription factor*, *MYB transcription factor*, *ZIP transporter protein*, and *Ring finger protein* in the homeostasis of Fe and Zn contents in plants (Tong et al., 2020). The QTL region *qFe_Ah03/qZn_Ah03* has corresponding eight candidate genes, such as *arahy.31BYXQ*, which encode a *zinc/iron permease* belonging to the *ZIP transporter family*; it is important for the transport of Fe and Zn contents for reserving their cellular homeostasis (Bouain et al., 2014; Guerinot, 2000; Kobayashi & Nishizawa, 2012), and it was highly expressed in leaves and pods. Furthermore, *arahy.AY0SR4* gene encodes a *bZIP transcription factor* and may be involved in zinc homeostasis during zinc‐deficient conditions (Assunção et al., 2010; Cifuentes‐Esquivel et al., 2018; Evens et al., 2017; Ishimaru et al., 2011). It exhibited higher expression in flowers and leaves. The TFs *bZIP19* and *bZIP23* were previously shown to regulate adaptation to zinc deficit in roots in *Arabidopsis thaliana* (Assunço et al., 2010; Inaba et al., 2015). In wheat, a total of 187 *TabZIP* genes were identified (Li et al., 2015), and some of these complement the function of zinc deficiency‐hypersensitive genes like *bzip19* and *bzip23* (Evens et al., 2017; Henriquez‐Valencia et al., 2018). Similarly, *arahy.Z9FA5A* gene encodes the *MATE efflux transporter*, which is responsible for transporting Fe from the root to the shoot (White & Broadley., 2009). It exhibits higher expression during seed developmental stages. Furthermore, *arahy.8KMA2D* gene, encoding the *vacuolar iron transporter* (VIT), plays an important role in storing and transporting Fe content into vacuoles (Connorton, Jones, et al., 2017). It shows high expression during seed developmental stage. Additionally, *arahy.42DQ0K* gene encodes the *SAUR‐like auxin‐responsive protein*, which plays an important role in the root morphology in response to Fe availability (Chen et al., 2010; Shen et al., 2015). Previously, AUX/IAA protein was found to impact seed Fe content in chickpeas (White & Broadley., 2009). Moreover, *arahy.E4I2JR* gene encodes the *bHLH 144 transcription factor*, which is involved in trace metal homeostasis and might play a role in the bio‐fortification of cereal grains with vital micronutrients like Zn (Menguer et al., 2018; Vatansever et al., 2017).

The QTL region *qFe‐Ah01*/*qZn‐Ah01* contains eight corresponding genes. For instance, *arahy.S59764* gene encodes the *vacuolar protein sorting associated protein 13*, responsible for seed Zn content (Dixit et al., 2019; Upadhyay et al., 2016). Additionally, *arahy.6AWP8P* gene encoding the *WD40 repeat* belonging to Zn finger family is responsible for zinc homeostasis (Bouain et al., 2014). Another gene *arahy.VE9WMU* encodes *MYB transcription* factor associated with Fe and Zn transports during nutrient deficiency (Shen et al., 2008). Similarly, *arahy.D92ENK* gene encodes the *cytochrome P450* and is found to be associated with Fe and Zn homeostases, with frequent expression observed under high Zn conditions (van de Mortel et al., 2006). This gene shows high expression in leaves. Furthermore, *arahy.QAC8GG* gene encodes *late embryogenesis abundant (LEA) protein* associated with high Fe and Zn contents in seed (Liu et al., 2011). It exhibits high expression during the seed developmental stage. Additionally, *Protein FAR1‐related sequence 3* is related to zinc ion binding. Recent studies reported FHY3/FAR1 are essential regulators of various physiological, developmental, and metabolic activities in several plant species in response to photoperiod (Li et al., 2011). Together, FHY3/FAR1 plays a significant role in the development of chloroplasts and the chlorophyll synthesis pathway during the early stages of seedling development (Tang et al., 2012).

Similarly, in QTL region *qFe‐Ah11*/*qZn‐Ah11*, plausible candidate genes were identified. The *arahy.FKL2A7*gene, which encodes the no apical meristem (NAC) domain, is of particular interest. *NAC transcription factor* was also found in wheat to enhance Fe and Zn contents (Uauy et al., 2006) and play a role in regulating the remobilization of Fe and Zn contents from source tissues to seeds during senescence (Ricachenevsky et al., 2013). This gene exhibits high expression during senescence. Additionally, *arahy.MH35TT* gene, encoding the *MYB domain*, plays an important role in Fe‐deficient conditions. It is a highly activated transcription factor that acts early in the Fe deficiency regulatory cascade to promote the expression of *NAS4* (Palmer et al., 2013). It shows high expression in flowers. According to Shen et al. (2008), MYB (*MxMYB1*) gene was isolated from *Malus xiaojinensis*, which is induced under Fe‐deficient conditions in roots. The expression of *MxMYB1* is upregulated by Fe‐deficient conditions in roots but not in leaves, suggesting its role in iron nutrition in roots.

Furthermore, QTL region *qZn‐Ah13* underpins two genes. The *arahy.H1181I* gene encodes *oxidoreductase activity*, and *arahy.4X60UW* gene encodes *SOUL heme binding protein*, which is important for iron binding. These genes exhibit high expression during pod development stage. Similarly, the QTL region *qFe_Ah07* corresponds to two genes: *arahy.1WFS8V*, encoding *Ring H2 finger protein* might be important for increasing Zn content and *arahy.VAAE0N* that encodes *protein kinase activity*. In the QTL region (*qZn_Ah08*), the *arahy.R4WH7M* gene encodes a *heavy metal transporter*. These identified candidate genes can undergo functional validation to elucidate their role in maintaining Fe and Zn homeostases.

## 5 CONCLUSION

The present study provides a comprehensive analysis of QTL mapping and candidate genes associated with Fe and Zn contents in groundnut. This study has identified nine major main‐effect QTLs and two main‐effect QTLs for Fe and Zn contents. Within these QTL regions, we have discovered a multitude of genes, including *ZIP transporter*, *bZIP transcription factor* and *VIT*, *WRKY transcription factor*, *vacuolar protein sorting associated protein*, *LEA protein*, and *bHLH transcription factor* genes. These genes are potential candidates that may play a crucial role in enhancing seed Fe and Zn contents.

Furthermore, these findings offer an opportunity for the functional validation of these candidate genes, enabling a deeper understanding of their specific contributions to Fe and Zn content regulations. By conducting further studies on these candidate genes, we can unravel the intricate pathways and processes that control the uptake, transport, and storage of Fe and Zn in groundnut, ultimately facilitating the development of groundnut varieties with enhanced nutritional value.

## AUTHOR CONTRIBUTIONS

**Sejal Parmar**: Data curation; formal analysis; investigation; methodology; software; validation; visualization; writing—original draft. **Pasupuleti Janila**: Data curation; investigation; methodology; resources; writing—review & editing. **Sunil S. Gangurde**: Data curation; formal analysis; investigation; methodology; software; validation; visualization; writing—review & editing. **Murali T. Variath**: Data curation; investigation; methodology; resources; validation; writing—review & editing. **Vinay Sharma**: Data curation; formal analysis; investigation; methodology; software; validation; visualization; writing—review & editing. **Deekshitha Bomireddy**: Data curation; formal analysis; investigation; methodology; software; validation; writing—review & editing. **Surendra S. Manohar**: Data curation; investigation; methodology; resources; validation; writing—review & editing. **Rajeev K. Varshney**: Methodology; resources; validation; writing—review & editing. **Prashant Singam**: Data curation; investigation; methodology; resources; validation; writing—review & editing. **Manish K. Pandey**: Conceptualization; data curation; funding acquisition; investigation; methodology; project administration; resources; software; supervision; validation; writing—original draft; writing—review & editing.

## CONFLICT OF INTEREST STATEMENT

The authors declare there is no conflict of interest.

## Supporting information

Table S1 List of epistatic QTLs for Fe and Zn contents for two seasons.
Table S2 Expression of candidate genes identified from QTL regions for 20 tissues for iron and zinc.

## Data Availability

The data sets generated/analyzed during current study are accessible from the corresponding author upon request.
